# Systemic Role for Vitamin D in the Treatment of Psoriasis and Metabolic Syndrome

**DOI:** 10.1155/2011/276079

**Published:** 2011-06-05

**Authors:** Lisa Wenyang Fu, Ronald Vender

**Affiliations:** ^1^Department of Medicine, McMaster University, 1200 Main Street West, Hamilton, ON, Canada L8N 3Z5; ^2^Dermatrials Research, Dermatology Centre, 132 Young Street, Hamilton, ON, Canada L8N 1V6

## Abstract

The novel discovery of the systemic role of vitamin D in the modulation of the immune system especially the Type 1 helper T cell (Th1) pathway reveals its potential for treating Th1 inflammatory diseases. Psoriasis has been recently established to be a systemic disease centered on inflammation and involvement of cytokines of the Th1 pathway. There is an increased prevalence of metabolic syndrome in patients with psoriasis. Metabolic syndrome also involves a proinflammatory state. This paper proposes the idea of the potential use of oral vitamin D to treat psoriasis and metabolic syndrome concurrently. We propose there is merit in more clinical trials investigating the use of vitamin D to treat both psoriasis and metabolic syndrome through its anti-inflammatory effects. On application to psoriasis management and prognosis, the goal is to decrease the risk for cardiovascular disease and decrease disease morbidity and mortality.

## 1. Introduction

The recent discovery that vitamin D receptors are found in most tissues and cells in the body opened a whole new arena of research. Vitamin D can play a role in decreasing the risk of many chronic illnesses, including autoimmune diseases, infectious diseases, cardiovascular disease, and common cancers such as colorectal, breast, and prostate cancers. It specifically has a role in cellular proliferation, differentiation, apoptosis, and angiogenesis [1]. Vitamin D has been found to be an immune regulatory hormone with beneficial effects on inflammatory diseases, mediated by helper T-lymphocytes type 1 (Th1) cells [2, 3], such as diabetes, psoriasis, Crohn's disease, and multiple sclerosis [1]. 

Psoriasis is a common Th1-mediated inflammatory disease characterized by scaly plaques on the skin, which can be painful and pruritic. It is also associated with psoriatic arthritis, Crohn's disease, diabetes mellitus (type 2), metabolic syndrome, depression, and cancer [4–6]. It affects 1–3% of the general population [4, 5].

Controversy exists over the precise criteria for diagnosis and classification of metabolic syndrome; however, it is accepted that it consists of a constellation of metabolic abnormalities including glucose intolerance, insulin resistance, central obesity, dyslipidemia, and hypertension [7]. The prevalence of metabolic syndrome varies greatly between populations and age groups ranging from 4% to 46% [8]. Metabolic syndrome is significantly increased in patients with psoriasis [9].

This paper strives to highlight the association between psoriasis, metabolic syndrome, and vitamin D. In addition, it proposes the hypothesis of potentially using oral systemic vitamin D as a modality to treat psoriasis and metabolic syndrome concurrently.

## 2. Psoriasis Pathophysiology

Psoriasis was first described as a disease that primarily affects epidermal keratinocyte proliferation and secondary cutaneous inflammatory infiltration [6]. In the last decade it has become evident that psoriasis is a systemic immune-mediated inflammatory disease primarily involving Th1 cells. Cytokines of the Th1 pathway (interferon-*γ*, interleukin 2, interleukin 12, and TNF-*α*) predominate in psoriatic plaques. It is widely accepted that an unknown stimulus activates cutaneous dendritic antigen-presenting cells. These activated antigen-presenting cells then activate helper T cells which lead to the subsequent release of a cascade of inflammatory cytokines. This cascade results in recruitment and activation of other cells types such as endothelial cells and neutrophils, and production of chemokines and growth factors. Eventually this leads to the proliferation of keratinocytes. A chronic inflammatory state then ensures and leads to the formation of psoriatic skin lesions [4, 6]. Recently, Interleukin-17-secreting helper T (Th17) cells have been identified to play an important role in psoriasis pathogenesis. Interleukin-17 promotes inflammation by inducing the expression of chemoattractants that are found in psoriatic lesions. Th17 cells also secrete interleukin 22, which is involved in keratinocyte differentiation retardation leading to keratinocyte proliferation [10].

## 3. Metabolic Syndrome Pathophysiology

Metabolic syndrome is accepted to be centered on insulin resistance and obesity. Free fatty acids (FFA) are released from abundant adipose tissue mass. FFA's effects on the liver include production of glucose and triglycerides, and secretion of very low density lipoproteins (VLDL) [7]. FFA inhibit insulin-mediated glucose uptake and therefore lead to insulin resistance. Increased circulating glucose and FFA increase pancreatic secretion of insulin resulting in hyperinsulinemia which can then increase sympathetic nervous system activity and contribute to hypertension [7]. Adipose tissue also contains cells such as adipocytes and monocyte-derived macrophages. These cells contribute to the proinflammatory state through the secretion of interleukin-6 (IL-6) and TNF-*α* among others [7]. These inflammatory factors lead to further insulin resistance and lipolysis of adipose tissue triglyceride stores and an additional increase in circulating FFA. There are also reductions in the production of adiponectin which is an anti-inflammatory and insulin sensitizing cytokine [7, 11]. The most widely accepted criteria for metabolic syndrome is by the National Cholesterol Education Program Adult Treatment Panel III. The definition defines metabolic syndrome as presence of at least three of the following: abdominal obesity (waist circumference equal to or greater than 102 cm in men; 88 cm in women), elevated serum triglycerides (equal to or greater than 150 mg/dL or drug treatment for elevated levels), low HDL cholesterol (men <40 mg/dL; women <50 mg/dL), and elevated blood pressure (equal to or greater than 130/85 mmHg or drug treatment for hypertension), elevated fasting glucose (equal to or greater than 110 mg/dL). The World Health Organization and the International Diabetes Foundation define metabolic syndrome under similar parameters [12, 13].

## 4. Psoriasis and Metabolic Syndrome

The pathogenesis of psoriasis and metabolic syndrome both involve inflammation. There is also evidence suggesting that there is a genetic link. A number of genes such as PSORS2, PSORS3, and PSORS4 are associated with psoriasis susceptibility and are also associated with metabolic disease [14]. Many studies have shown a link between psoriasis and metabolic syndrome [14]. To highlight, Gisondi and colleagues [9] established that there is a 30.1% prevalence of metabolic syndrome in psoriatic patients compared to 20.6% in the control population (*P* = .005, OR: 1.65, 95%, confidence interval: 1.16–2.35). Sommer and colleagues [13] reported that German patients hospitalized for psoriasis were 6-fold more likely to have metabolic syndrome compared with control patients admitted for melanoma surgery. Few studies have explored the possibility of treating psoriasis by treating components of metabolic syndrome. Naldi and colleagues [15] showed that in a large-scale cohort study of 2000 patients, obese patients had more severe psoriasis that were more resistant to treatment compared to non obese psoriatic patients. Hossler and colleagues [16] observed two patients with body mass indices greater than 50 kg/m^2^ who had marked improvement in their psoriasis after gastric bypass surgery and weight loss.

## 5. Cardiovascular Disease Risk

Cardiovascular disease, like psoriasis and metabolic syndrome, also result from a proinflammatory state. Endothelial cells in atherosclerotic blood vessels facilitate the attachment of T-lymphocytes, the attraction of mast cells, and, as a result, the release of a cascade of proinflammatory cytokines such as TNF-*α* is initiated. The proinflammatory state plays a key role in fatty streak formation, plaque formation, and eventually thrombosis [17]. Studies have shown an increased risk of myocardial infarction (MI) and stroke in patients with psoriasis. Gelfand and colleagues [18] showed that 2.9% of patients in the severe psoriasis group developed MIs while only 2.0% of the control population developed MIs. Gelfand and colleagues [19] also found that there is a 50% increase in mortality in patients with severe psoriasis compared to the control group. They found that patients with severe psoriasis died 3.5 (male) and 4.3 (female) years younger than patients without psoriasis. Mehta and colleagues [20] found that patients with severe psoriasis have a clinically significant 57% increased risk of cardiovascular death while adjusting for conventional cardiovascular risk factors (history of MI, stroke, transient ischemic attack, or atherosclerotic disease). This suggests that psoriasis is an independent risk factor for cardiovascular disease. 

Similarly, patients with metabolic syndrome are also at an increased risk for cardiovascular disease. Adipose tissue overproduces plasminogen activator inhibitor-1 (PAI-1). In addition, cytokines and FFA in metabolic syndrome also increase the liver production of fibrinogen and PAI-1. The increase in PAI-1 and fibrinogen results in a prothrombotic state. Studies have shown increased risk for cardiovascular disease and mortality in patients with metabolic syndrome [21]. Isomaa and colleagues [22] found that subjects with metabolic syndrome were at a three-fold increased risk for developing coronary heart disease and stroke (*P* < .001). Cardiovascular mortality was also markedly increased in patients with metabolic syndrome (12.0%) compared to controls (2.2%; *P* < .001). Lakka and colleagues [23] reported that men with metabolic syndrome were 2.9–4.2-times more likely to die from cardiovascular disease compared to controls after adjustment for conventional cardiovascular risk factors. 

Therefore patients with both psoriasis and metabolic syndrome are at a greatly increased risk for developing cardiovascular disease.

## 6. Vitamin D and Psoriasis

Vitamin D has been used to treat psoriasis in the topical form with great success [24, 25]. 1-*α*, 25-dihydroxyvitamin D3 (calcitriol) is the hormonally active form of Vitamin D. It affects cellular function by acting through the vitamin D receptor (VDR) on keratinocytes [26, 27]. VDR binds to and activates transcription of genes that influence growth, differentiation, and inflammation in keratinocytes. Calcitriol has also been shown to have immunomodulatory effects on monocytes, macrophages, T cells, and dendritic cells [26, 27]. It is believed that through these mechanisms topical Vitamin D actively treats psoriatic skin lesions. However, the evidence that psoriasis is a systemic disease, which affects many organ systems, and involves many comorbidities, namely, the cardiovascular system suggests merit in revisiting systemic oral vitamin D for treatment of the inflammatory pathogenesis of psoriasis. At this point in time, there are very few studies that have investigated the use of oral vitamin D in patients with psoriasis. Perez and colleagues [28] established that 88% of 85 psoriasis patients treated with oral vitamin D had improvement in their psoriasis, 26.5% had complete clearance, 36.2% had moderate improvement, and 25.3% had slight improvement. An evaluation of serum calcium concentrations and urinary calcium excretion and creatinine clearance suggested that oral Vitamin D altered creatinine metabolism or secretion but did not affect renal function [28]. Werner de Castro and colleagues [29] published the only report to date of the resolution of anti-TNF*α*-induced psoriasiform lesions (biopsy confirmed) by doses of Vitamin D3 in a patient with vitamin D deficiency and rheumatoid arthritis. The recent discovery of the systemic role of vitamin D suggests that there is great merit in revisiting the use of system vitamin D to treat psoriasis with large scale clinical trials to assess the safety and efficacy. It would also be very interesting and relevant to conduct studies looking at the baseline serum vitamin D level in patients with psoriasis.

## 7. Vitamin D and Metabolic Syndrome

Recent research suggests that vitamin D can improve metabolic syndrome. Vitamin D has been proposed to be sequestered in the abundance of adipose tissue in metabolic syndrome, with decreased circulating levels [30]. Vitamin D deficiency in obese patients is further increased due to decreased sun exposure because of reduced mobility and wearing clothing that covers most areas of skin because of cosmetic preference. Vitamin D reduces inflammation by modulating the expression of several cytokine genes [31]. Tzotzas and colleagues [32] reported rising serum vitamin D levels after weight loss in obese women. Chui and colleagues [33] found that subjects with hypovitaminosis have a higher risk of insulin resistance and metabolic syndrome. Alvarez and Ashraf [34] found in their meta-analysis of both cross-sectional and prospective studies that vitamin D insufficiency (20–29 ng/ml) and deficiency (less than 20 ng/ml) have direct and indirect effects on insulin secretion and insulin action. Maki and colleagues [35] found that serum triglycerides, waist circumference, and body mass index is inversely related to vitamin D levels. They showed that the prevalence of metabolic syndrome is inversely proportional to serum vitamin D levels, suggesting that there is a link between metabolic syndrome and lower vitamin D levels. Future studies need to be performed to assess controlled supplementation of vitamin D and its effects on components of metabolic syndrome [36]. 

In addition, low vitamin D levels have recently been associated with an increased incidence of cardiovascular events [37, 38].

## 8. Conclusion

In conclusion, metabolic syndrome and psoriasis are closely related and share common genetic and inflammatory components. As discussed above there has been recent research suggesting vitamin D plays a role in metabolic syndrome and improves psoriatic skin lesions. We propose that there is merit in performing large-scale clinical trials aimed at revisiting the use of oral vitamin D to directly target manifestations of psoriasis and metabolic syndrome at the same time. On application to psoriasis management and prognosis, the use of oral vitamin D has great potential in clearing psoriatic skin lesions and at the same time also decreasing the risk for cardiovascular disease and decreasing disease morbidity and mortality. Randomized, blinded, large-scale, and long-term clinical studies are needed to address this important issue.
